# Extracapsular Dissection of Deep Lobe Parotid Mass Aided by Retromandibular Vein on Imaging: A Case Report

**DOI:** 10.7759/cureus.38874

**Published:** 2023-05-11

**Authors:** Basem Jamal

**Affiliations:** 1 Oral and Maxillofacial Surgery, King Abdulaziz University, Jeddah, SAU; 2 Oral and Maxillofacial Surgery, King Abdullah Medical City, Makkah, SAU

**Keywords:** facial nerve injury, retromandibular vein, parotid mass, deep lobe, ecd, extracapsular dissection

## Abstract

This report investigates the use of the retromandibular vein on imaging as a diagnostic tool for planning deep lobe parotid tumors. A unique aspect of this case is the performance of extracapsular dissection on a deep lobe parotid lesion, which is rare. Preoperative imaging showed a superficially displaced retromandibular vein, indicating a deeply seated tumor, which aided surgical planning. Under general anesthesia, extracapsular dissection was performed while protecting the facial nerve branches. The patient's postoperative course was uneventful, and the facial nerve was intact with no weakness.

## Introduction

Deep lobe parotid tumors are rare, and they account for approximately 20% of all parotid tumors. Surgical excision is the most commonly used treatment for parotid tumors, but it carries the risk of facial nerve injury. Such injuries can range from minor and temporary to more severe and permanent nerve damage [1]. Compared to superficial parotidectomy, deep lobe parotidectomy has been associated with a significantly higher incidence of facial nerve injury in several studies [2, 3]. According to a recent meta-analysis of deep lobe parotid tumors, 2.6% of patients experienced permanent postoperative facial nerve injury, while 32.5% experienced transient injury [4].

Extracapsular dissection (ECD) in parotid surgery refers to a surgical technique used to remove a clinically benign parotid mass in which the surgeon removes the tumor while leaving the capsule or outer layer of the gland intact, which helps to preserve the function of the gland and reduce the risk of facial nerve damage and ECD is generally considered a safe and effective surgical technique for the removal of clinically benign parotid gland masses. However, the procedure may pose challenges and risks when dealing with deep-seated tumors. Historically, ECD was primarily indicated for masses in the superficial lobe [5-7].

Here, we describe a case of using ECD to remove a deep parotid tumor, and we also discuss the preoperative localization of the facial nerve through imaging diagnostic tools, particularly the retromandibular vein.

## Case presentation

**Figure 1:**
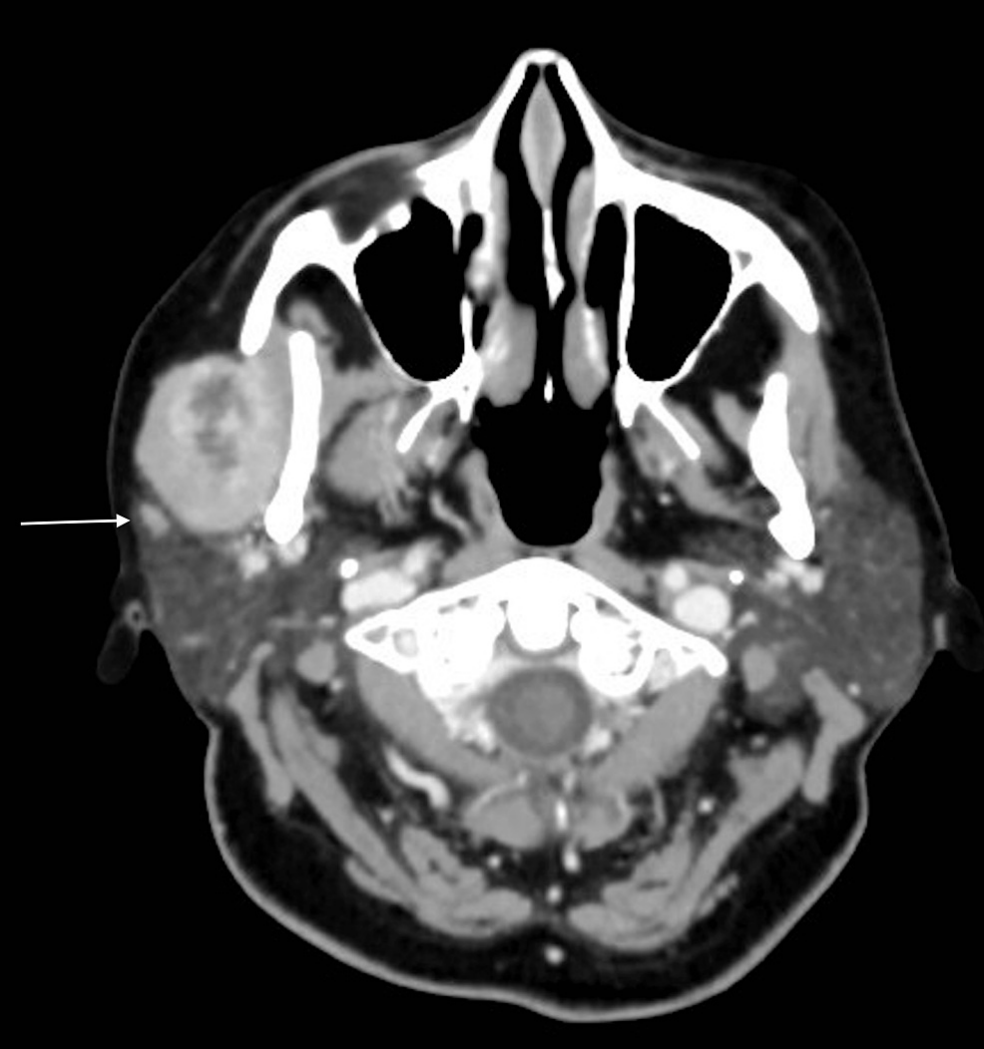
Preoperative neck CT scan A neck CT scan showed a well-circumscribed heterogeneously enhancing lesion with central hypodensity measuring 3X2.7X3.5 cm. The white arrow is pointing to the retromandibular vein.

A 42-year-old female patient was referred to the department of oral and maxillofacial surgery for the management of a parotid mass. The patient reported that she first noticed the mass 10 years ago, and it has been slowly growing since then. She denied experiencing any other symptoms, such as fever, pain, numbness, or facial weakness. Upon clinical examination, a mobile, firm mass was observed in the right upper neck area, measuring 2x3cm, with intact overlying skin. No associated cervical lymphadenopathy was present, and the cranial nerve examination was normal bilaterally, with no altered sensation at the auricular area or weakness of the facial expression muscles. The mucosa of the oral cavity appeared normal with no signs of ulcerations, and no masses were observed in the oropharynx upon examination. The neck CT scan displayed a well-defined lesion that showed heterogeneous enhancement with a central hypodensity, measuring 3 x 2.7 x 3.5 cm (Figure 1). However, the report did not mention the retromandibular vein, and it indicated that the mass was superficial.

The patient had a fine needle aspiration of the mass, which revealed serous-type acinar cells that had a positive DOG1 stain, raising the possibility of acinar cell carcinoma. The surgical plan was to remove the parotid mass using the extracapsular dissection technique, and the patient was extensively counseled about the potential risk of facial nerve injury and its associated consequences.

**Figure 2:**
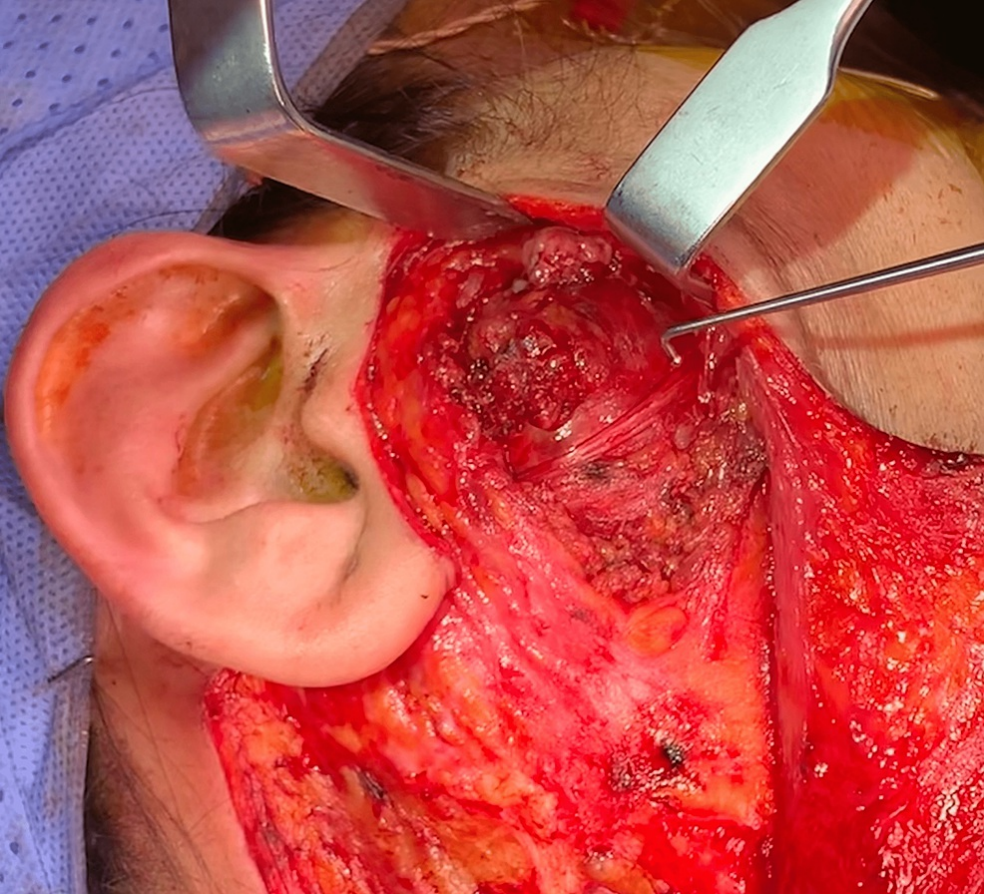
Parotid mass during extracapsular dissection Extracapsular dissection of the deeply seated parotid mass showing the overlying facial nerve. The nerve hook points to the buccal branch of the facial nerve.

The surgical approach involved making a modified Blair incision in the preauricular skin crease, starting above the tragus and extending down to the ear lobule. The incision was then extended posteriorly to the postauricular area and inferiorly to a natural horizontal crease in the neck. An anterior skin flap was subsequently raised in a subplatysmal plane as well as in the plane between the superficial muscular aponeurotic system (SMAS) layer and the parotid fascia to expose the parotid mass situated beneath the parotid fascia. A vertical incision was created in the parotid capsule, followed by careful blunt dissection under loupe magnification and nerve monitoring to identify the facial nerve. Sharp and blunt dissection was continued until the buccal branch of the facial nerve (nerve hook in Figure 2), temporal branch, and marginal mandibular branch were identified.

**Figure 3:**
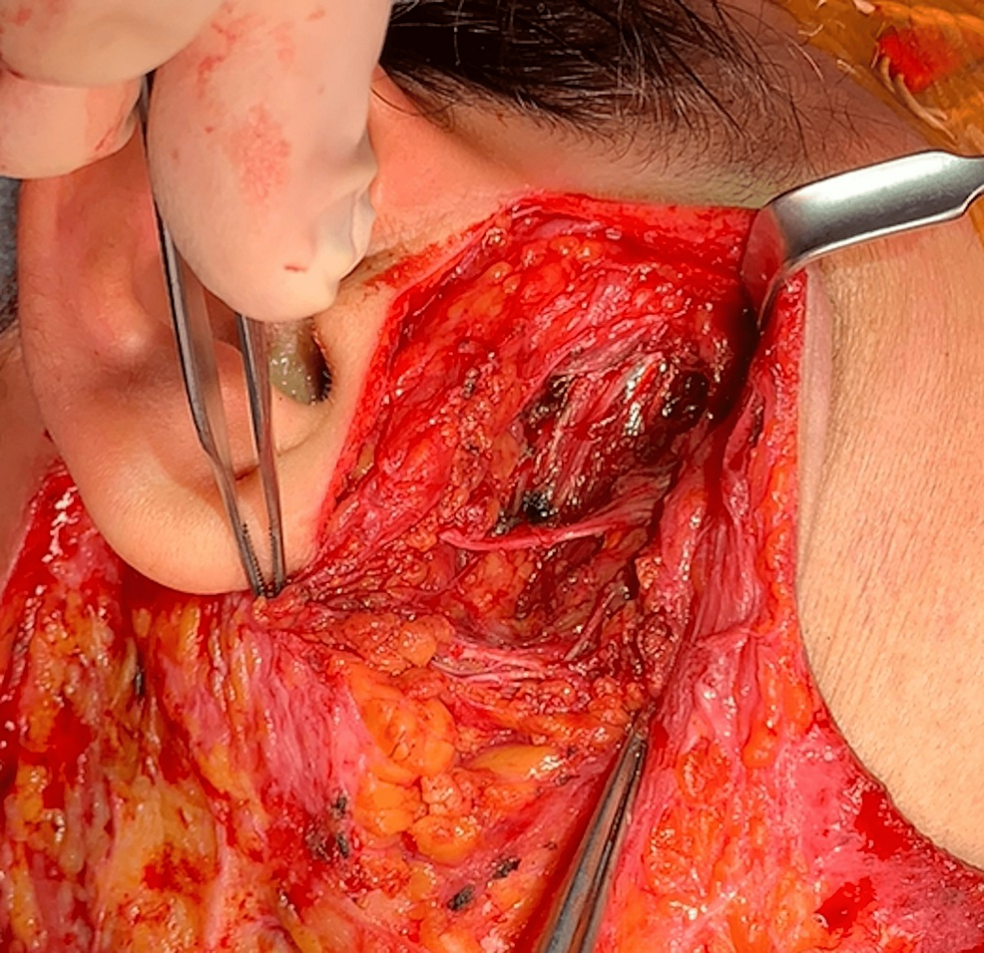
Parotid defect following extracapsular dissection Defect revealing intact buccal branch of the facial nerve following excision of the parotid mass via extracapsular dissection.

After releasing the nerve from the tumor, extracapsular dissection of the deep lobe parotid tumor was performed, and the mass was completely excised. At the end of the procedure, all nerve branches were found to be intact (Figure 3).

**Figure 4:**
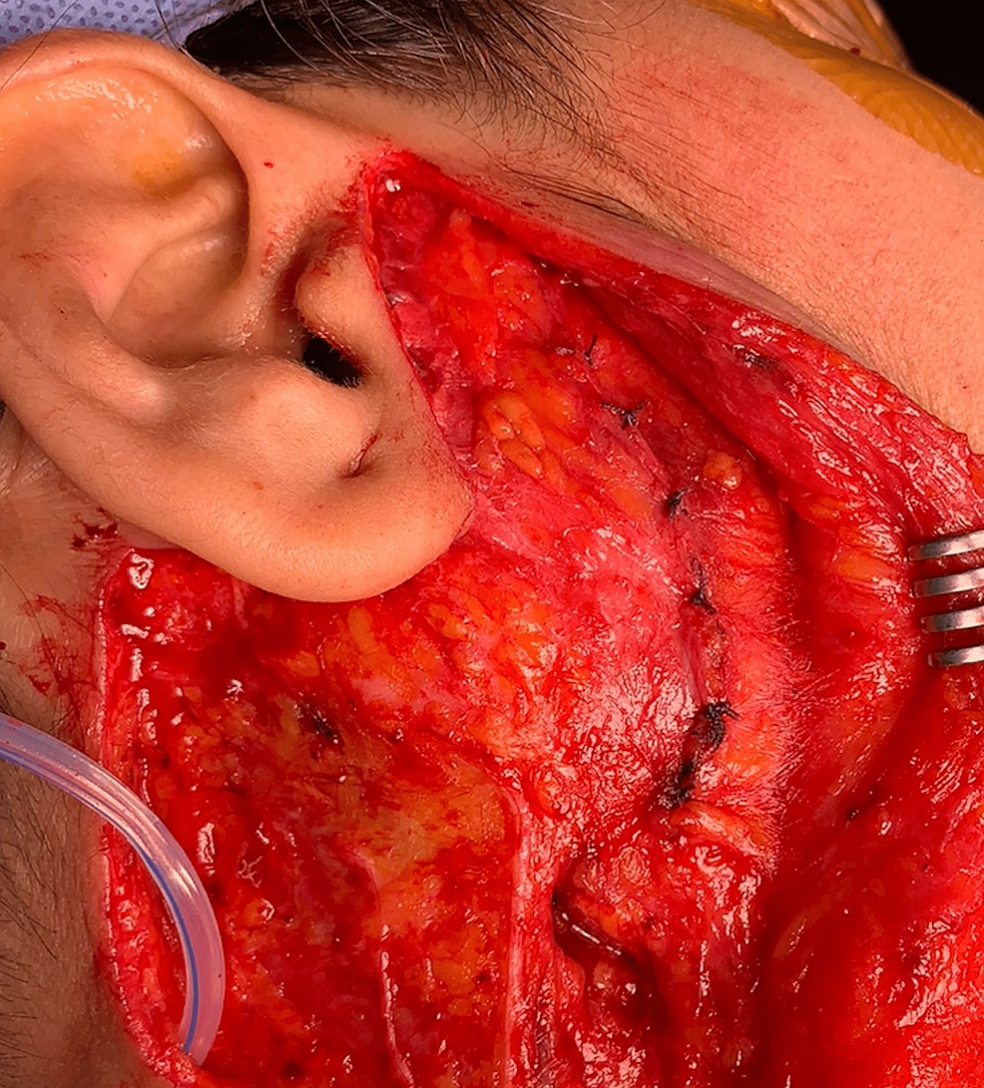
Parotid fascia closure following extracapsular dissection

The parotid fascia was sutured using 40 Vicryl, and the skin flap was reapproximated (Figure 4).

The patient's postoperative course was uneventful and without any complications. The wound showed satisfactory healing without any signs of infection or erythema, and the function of the facial nerve was preserved with no signs of weakness. The final pathology was confirmed to be acinic cell carcinoma.

## Discussion

Although the majority of parotid tumors are found in the superficial lobe, about 20% are located in the deep lobe. Extracapsular dissection (ECD) has been found to be a similarly effective surgical option as superficial parotidectomy in terms of tumor control and recurrence rates. In addition, ECD has been associated with fewer postoperative complications and is now widely regarded as the preferred surgical technique for clinically benign parotid tumors [8]. Albergotti et al. reported a significantly lower incidence of Frey’s syndrome and facial nerve paresis following ECD compared to superficial parotidectomy [6]. Furthermore, studies have also shown the efficacy of ECD as the sole treatment for low-grade malignant parotid lesions [9]. However, ECD is not without limitations. That includes tumor size, as reports used a tumor size cutoff between 2.5 and 4 cm because ECD for larger tumors was found to be associated with a higher risk of facial nerve injury [6]. Various diagnostic imaging modalities have been researched to assist in preoperative planning for parotid surgery [10,11]. The presented case displayed typical clinical and radiological characteristics of a benign salivary gland condition, which is mostly found in the superficial lobe. Although the radiology report indicated that the lesion was superficial and did not mention the retromandibular vein's position in relation to the parotid mass, the vein's location was the only indication of the tumor being located deep to the facial nerve. The parotid gland is divided into superficial and deep lobes by the retromandibular vein, which is located within the gland. The extracranial part of the facial nerve is situated just laterally to this vein, making it a crucial reference point for surgical planning [12]. It has been demonstrated that the retromandibular vein can serve as an effective diagnostic tool for predicting the location of a parotid tumor. However, the accuracy of this method may vary depending on the patient and the characteristics of the tumor. Several studies have reported different outcomes regarding the reliability of this landmark in predicting the location of a parotid tumor [13,14]. The incidence of facial nerve injury after parotid surgery is relatively high, ranging from 10% to 30%, with even higher rates in patients who undergo total parotidectomy. A meta-analysis conducted by Chen et al. revealed an overall facial nerve paralysis rate of 11% for patients with superficial lobe tumors and 40% for patients with deep lobe tumors [15]. Patients who undergo surgical management for deep lobe parotid tumors have a significantly higher rate of postoperative facial nerve injury, with an overall injury rate of 35.1 %, compared to patients with superficial lobe tumors who undergo parotidectomy, which has a transient injury rate of 11.7% [7].

## Conclusions

This case study highlights the success of using ECD for treating deep lobe tumors without increasing the risk of facial nerve paresis. It also emphasizes the significance of including information about the retromandibular vein in any imaging related to parotid tumors to assist in surgical planning for parotid gland procedures.
